# Impacto da Inovação Tecnológica no Tratamento e Prognóstico da Insuficiência Cardíaca

**DOI:** 10.36660/abc.20240680

**Published:** 2024-11-08

**Authors:** João Marcos Bemfica Barbosa Ferreira, Maria Helena Costa de Vasconcelos, Andreza Araújo de Oliveira, Maria Eduarda da Silva Corrêa

**Affiliations:** 1 Universidade do Estado do Amazonas Manaus AM Brasil Universidade do Estado do Amazonas, Manaus, AM – Brasil; 2 Universidade Nilton Lins Manaus AM Brasil Universidade Nilton Lins, Manaus, AM – Brasil

**Keywords:** Insuficiência cardíaca, Epidemiologia, Prognóstico

O artigo intitulado " Avaliação da Congestão Pulmonar por Ultrassom e Sensoriamento Dielétrico Remoto (ReDS) em Pacientes Hospitalizados com Insuficiência Cardíaca"^1^ nos traz uma importante contribuição para a adequada avaliação de pacientes portadores de insuficiência cardíaca (IC) que apresentem congestão pulmonar, a qual representa uma frequente causa de descompensação e internação em pacientes com IC.

A IC, tanto com fração de ejeção preservada quanto reduzida, tem elevada prevalência, com aumento devido ao envelhecimento da população.^2^ Além disto apresenta alta mortalidade mesmo com o advento de novas drogas e novos alvos terapêuticos.^3^

O prognóstico da IC pode ser avaliado de várias formas, incluindo a classe funcional (NYHA) e parâmetros ecocardiográficos através de biomarcadores, como os peptídeos natriuréticos. Outro ponto importante a ser considerado é a avaliação etiológica da doença, uma vez que o prognóstico também difere entre as diversas etiologias. Ademais, outros fatores devem ser considerados, incluindo dados de história clínica, exame físico, exames complementares, avaliação hemodinâmica e tolerância às medicações com impacto em mortalidade.^4^ Dessa forma, buscando diminuir a subjetividade durante a avaliação prognóstica do paciente com IC, alguns escores de riscos foram criados. Dentre eles, os mais utilizados na prática clínica são o *Heart Failure Survival Score* (HFSS) e o *Seattle Heart Failure Model* (SHFM).^5^ Ademais, dentre as principais causas de descompensação associadas a IC, a doença renal crônica (DRC) é uma das principais, visto que com muita frequência DRC e IC coexistem, além de compartilharem diversos fatores de risco, culminando com um pior prognóstico aos pacientes.^6^ Em pacientes com IC as descompensações representam um importante fator prognóstico causando frequentes internações que aumentam sua mortalidade. Entre as principais causas de descompensação encontra-se a má adesão medicamentosa e o estilo de vida.^7^ Entre todas as causas de descompensação a congestão pulmonar está presente na maioria dos casos e sua detecção, principalmente quando precoce, pode diminuir a chance de internação e, consequentemente melhorar a qualidade de vida e prognóstico dos pacientes. Técnicas inovadoras têm buscado identificar pacientes propensos à descompensação, principalmente a nível extra-hospitalar.

A inovação tecnológica na IC está transformando a forma como os pacientes são monitorados em relação às descompensações. Dessa forma, dispositivos implantáveis, como monitores hemodinâmicos, têm mostrado bom desempenho na melhora da sobrevida em pacientes com fração de ejeção reduzida, pois eles fornecem dados contínuos sobre a pressão arterial e outros parâmetros cardíacos.^8^ Além disso, existem balanças inteligentes combinadas com biomarcadores digitais que oferecem uma abordagem não invasiva para monitorar a função cardíaca, através da medição do volume sanguíneo, redistribuição de líquidos corporais e função cardíaca – como o débito cardíaco. Isso é possível devido a tecnologias como o balistocardiograma e a impedância bioelétrica, que conseguem detectar as mudanças na circulação sanguínea e na composição corporal com base na condutividade elétrica do corpo.^9^ Ademais, as estratégias de monitoramento remoto estão evoluindo com o uso de algoritmos que avaliam múltiplos dados do paciente em tempo real, permitindo uma coleta contínua de dados fisiológicos, como pressão arterial, frequência cardíaca e níveis de oxigênio. Com isso, a intervenção é rápida e personalizada, reconhecendo mudanças sutis que precedem a descompensação, reduzindo hospitalizações e melhorando a qualidade de vida dos pacientes. Além disso, os dados dos pacientes podem ser acompanhados pela equipe médica através de aplicativos móveis e plataformas de saúde digital, permitindo ajuste terapêutico de forma precoce, como também melhora na adesão ao tratamento e acompanhamento regular do paciente.^10^

Logo, as inovações tecnológicas estão promovendo um novo paradigma no cuidado de pacientes com IC, focando em abordagens mais proativas e preventivas, o que apresenta um grande potencial de melhora na qualidade de vida e prognóstico destes pacientes.
