# Supplementary material for: Impacto da Inovação Tecnológica no Tratamento e Prognóstico da Insuficiência Cardíaca
Source: Arq Bras Cardiol. 2024 Nov 8;121(10):e20240680. [Article in Portuguese] doi: 10.36660/abc.20240680 (PMC11634211; doi:10.36660/abc.20240680)
Supplement: Translation

# Translation: Impact of Technological Innovation in the Treatment and Prognosis of Heart Failure

**DOI:** 10.36660/abc.20240680i

**Authors:** João Marcos Bemfica Barbosa Ferreira, Maria Helena Costa de Vasconcelos, Andreza Araújo de Oliveira, Maria Eduarda da Silva Corrêa

**Affiliations:** 1 Manaus AM Brazil Universidade do Estado do Amazonas, Manaus, AM – Brazil; 2 Manaus AM Brazil Universidade Nilton Lins, Manaus, AM – Brazil

**Keywords:** Heart Failure, Epidemiology, Prognosis

The manuscript entitled "Assessment of Pulmonary Congestion According to Ultrasound and Remote Dielectric Sensing (ReDS) in Patients Hospitalized With Heart Failure"^1^ brings us an important contribution to the adequate evaluation of patients with heart failure (HF) who present pulmonary congestion, which represents a frequent cause of decompensation and hospitalization in patients with HF.

HF, both with preserved and reduced ejection fraction, has a high prevalence, with an increase due to the aging of the population.^2^ Furthermore, it presents high mortality even with the advent of new drugs and new therapeutic targets.^3^

The prognosis of HF can be assessed in several ways, including the functional class (NYHA), echocardiographic parameters, through biomarkers, such as natriuretic peptides. Another important point to be considered is the etiological assessment of the disease, since the prognosis also differs between different etiologies. Furthermore, other factors must be considered, including data from clinical history, physical examination, complementary tests, hemodynamic assessment and tolerance to medications with an impact on mortality.^4^ Therefore, seeking to reduce subjectivity during the prognostic assessment of patients with HF, some risk scores were created. Among them, the most used in clinical practice are the Heart Failure Survival Score (HFSS) and the Seattle Heart Failure Model (SHFM).^5^ Furthermore, among the main causes of decompensation associated with HF, chronic kidney disease (CKD) is one of the main ones, since CKD and HF very often coexist, in addition to sharing several risk factors, culminating in a worse prognosis for patients.^6^ In patients with HF, decompensation represents an important prognostic factor, causing frequent hospitalizations that increase mortality. Among the main causes of decompensation is poor medication adherence and lifestyle habits.^7^ Among all the causes of decompensation, the presence of pulmonary congestion is present in most cases and its detection, especially when early, can reduce the chance of hospitalization and, consequently, improving the quality of life and prognosis of patients. Innovative techniques have sought to identify patients prone to decompensation, especially at an out-of-hospital level.

Technological innovation in HF is transforming the way patients are monitored for decompensation. Therefore, implantable devices, such as hemodynamic monitors, have shown good performance in improving survival in patients with reduced ejection fraction, as they provide continuous data on blood pressure and other cardiac parameters.^8^ Additionally, there are smart scales combined with digital biomarkers that offer a non-invasive approach to monitoring cardiac function by measuring blood volume, body fluid redistribution and cardiac function – such as cardiac output. This is possible due to technologies such as bioelectrical impedance, which can detect changes in blood circulation and body composition based on the body's electrical conductivity.^9^ Furthermore, remote monitoring strategies are evolving with the use of algorithms that evaluate multiple patient data in real time, allowing continuous collection of physiological data, such as blood pressure, heart rate and oxygen levels. Therefore, the intervention is quick and personalized, recognizing subtle changes that precede decompensation, reducing hospitalizations and improving patients’ quality of life. Furthermore, patient data can be monitored by the medical team through mobile applications and digital health platforms, allowing early therapeutic adjustment, as well as improving treatment adherence and regular patient monitoring.^10^

Therefore, technological innovations are promoting a new paradigm in the care of patients with HF, focusing on more proactive and preventive approaches, which has great potential for improving the quality of life and prognosis of these patients.
